# Association of Baseline Femoral Trochlear T2* Mapping with Clinical Response to Platelet-Rich Plasma in Patellofemoral Chondropathy: A Retrospective Exploratory Study

**DOI:** 10.3390/jcm15114324

**Published:** 2026-06-03

**Authors:** Carla Fuster Such, Francisco Lajara-Marco, Jorge Salvador-Marín, Vicente J. León-Muñoz, María Francisca Cegarra-Navarro

**Affiliations:** 1 Department of Radiology, Hospital Universitario del Vinalopó, 03293 Elche, Spain; carlafustersuch@gmail.com; 2 Department of Orthopaedic Surgery and Traumatology, Hospital General Universitario Reina Sofía, 30003 Murcia, Spain; vleonmd@gmail.com; 3 Instituto Murciano de Investigación Biosanitaria Pascual Parrilla (IMIB), 30120 Murcia, Spain; 4 Department of Orthopaedic Surgery and Traumatology, Hospital General Universitario Dr. Balmis, 03010 Alicante, Spain; 5 Department of Radiology, Hospital General Universitario Reina Sofía, 30003 Murcia, Spain

**Keywords:** platelet-rich plasma, PRP, patellofemoral chondropathy, T2* mapping, magnetic resonance imaging, cartilage, candidate prognostic biomarker, reproducibility, Kujala, VAS

## Abstract

**Background:** Platelet-rich plasma (PRP) is utilised in the treatment of patellofemoral chondropathy, although clinical responses remain variable. This retrospective exploratory study assessed whether baseline quantitative T2* mapping of femoral cartilage was associated with clinical improvement following PRP administration. **Methods:** In this retrospective observational study conducted within routine clinical practice, patients with patellofemoral chondropathy received three ultrasound-guided intra-articular PRP injections administered weekly according to an institutional protocol. Baseline and 9-month T2*-mapping MRI scans and clinical questionnaires were collected as part of standard follow-up. The main imaging variable was the worst-region femoral trochlear T2* value, evaluated as a candidate prognostic biomarker. Clinical outcomes included the Visual Analogue Scale (VAS, 0–10) and Kujala (0–100) scores, with responders defined by minimum clinically important difference (MCID) thresholds (ΔVAS ≥ 1.5; ΔKujala ≥ 8). **Results:** Thirty-two knees from 22 patients completed follow-up, including 10 bilateral cases (19 right knees, 13 left knees). Both VAS and Kujala scores improved significantly at 9 months (*p* < 0.001 for both). Baseline femoral trochlear worst-region T2* values were inversely correlated with pain and functional improvement (ΔVAS: rho = −0.51, *p* = 0.003; ΔKujala: rho = −0.36, *p* = 0.042). Baseline patellar T2* values were not associated with clinical change (ΔVAS: rho = −0.18, *p* = 0.32; ΔKujala: rho = −0.12, *p* = 0.51). Sensitivity analyses using baseline mean femoral T2* values did not show significant associations with ΔVAS or ΔKujala. Interobserver reproducibility for the worst-region T2* metric was limited, particularly for the femoral compartment (femur ICC 0.37; patella ICC 0.47), which limits immediate clinical applicability. Mean regional longitudinal ΔT2* changes did not exceed the 14% QIBA MDC95 threshold. **Conclusions:** In this small retrospective cohort, baseline femoral trochlear worst-region T2* values were associated with clinical improvement after PRP. These preliminary hypothesis-generating findings should be interpreted with caution and require validation in larger controlled cohorts with standardised and reproducible segmentation workflows.

## 1. Introduction

Patellofemoral chondropathy is a common cause of anterior knee pain and functional limitation, especially among young, physically active individuals. Intra-articular platelet-rich plasma (PRP) is increasingly utilised as a biologic therapy for patellofemoral cartilage-related symptoms; however, clinical response remains heterogeneous across studies and patient populations. This variability highlights the need for robust predictors to inform patient selection and manage expectations [1].

Conventional morphological MRI is essential for depicting focal cartilage defects and coexisting structural abnormalities; however, morphological sequences may not fully capture early or “pre-morphologic” alterations in cartilage matrix composition. Quantitative T2* mapping is a compositional (“molecular”) MRI technology that provides voxel-wise relaxation times reflecting cartilage water content and collagen network organisation. These properties make T2* mapping a non-invasive biomarker candidate for detecting early cartilage matrix degeneration and for monitoring matrix response to interventions [2,3].

The technical performance and interpretability of quantitative cartilage mapping have improved through standardisation initiatives. The Quantitative Imaging Biomarkers Alliance (QIBA) profile for cartilage compositional imaging establishes reproducibility targets, suggesting that approximately 14% change in T2* (or T1ρ) represents the minimum detectable change with 95% confidence. When only increases are anticipated over time, approximately 12% change may be considered the minimum detectable threshold [4]. These benchmarks support the use of T2* mapping in longitudinal studies and clinical trials to detect subtle matrix changes beyond conventional morphology [4].

In patellofemoral cartilage specifically, T2* mapping has been used to characterise patellar chondromalacia and to correlate quantitative compositional measures to morphological grading [3]. Beyond cross-sectional characterisation, axial T2* mapping at 3T has shown potential to quantify and predict progression in untreated patellar cartilage defects over multi-year follow-up [5], and quantitative T2* mapping has also been incorporated into imaging evaluation frameworks after cartilage repair to assess graft viability and integration alongside conventional MRI [6].

Evidence linking PRP to compositional cartilage imaging endpoints is emerging. In a patellofemoral PRP cohort combining clinical outcomes with quantitative mapping, T2* mapping was used to evaluate patellofemoral cartilage after intra-articular PRP injections [7]. In addition, in a randomised placebo-controlled injection trial in mild-to-moderate knee osteoarthritis, quantitative mapping (including T2 and T2*) and morphological MRI were used to compare cartilage changes after PRP and other injectables versus placebo, supporting the feasibility of MRI mapping as a structural endpoint in injection studies [8].

Despite this promise, quantitative compositional biomarkers do not consistently correlate with pain or function, and their current role in routine clinical decision-making remains debated; they are best interpreted alongside clinical outcomes and morphology, and further work is needed to establish clinically actionable thresholds and standardised workflows [9].

Therefore, the primary exploratory objective of this study was to evaluate whether baseline quantitative T2* values of the femoral trochlea (worst region) were associated with clinical response to PRP in patellofemoral chondropathy, as measured by changes in pain (ΔVAS) and function (ΔKujala). Secondary objectives were to explore patellar T2* metrics, short-term T2* changes after treatment, qualitative grading on T2* maps, and interobserver agreement between two independent readers. Given the retrospective design and the inherent reproducibility concerns of focal worst-region measurements, these analyses were considered preliminary and hypothesis-generating.

## 2. Materials and Methods

### 2.1. Study Design, Setting, and Ethics

This single-centre retrospective observational study utilised clinical and imaging data collected during routine practice from patients with symptomatic patellofemoral chondropathy treated with intra-articular platelet-rich plasma (PRP). All patients were managed according to the institutional clinical protocol entitled “PRP infiltration protocol for patellar chondropathy, Musculoskeletal Radiology Unit, HGURS,” which was implemented in July 2021. For the present research project, the cohort was retrospectively defined from patients who had received PRP between January 2022 and January 2023 and who had complete baseline and protocol-based 9-month MRI and clinical follow-up; consequently, follow-up data collection for the analysed cohort extended until October 2023. The decision to analyse these routine-care data for research purposes was made after the data had already been collected. The project was initially processed through our institution in October 2022 and, because PRP is considered a medicinal product in Spain, it was referred to the CEIm Hospital Virgen de la Arrixaca, which approved this retrospective analysis as an observational study with medicinal products (protocol number 2022-11-5-HCUVA; protocol version 1.1 dated 20 January 2023; approval date 28 February 2023).

### 2.2. Patient Selection and Eligibility Criteria

Patients were identified in the outpatient setting among adults (older than 18 years old) with anterior knee pain attributed to patellofemoral chondropathy who had persistent symptoms despite at least 6 months of conservative management, including NSAIDs, chondroprotective agents, and supervised physiotherapy, and who were considered candidates for PRP therapy in routine clinical practice between January 2022 and January 2023. As part of the clinical pathway, patients received the corresponding information sheet and signed written consent for the PRP procedure and scheduled follow-up assessments. For the present retrospective analysis, only knees with available baseline MRI, including quantitative T2* mapping, and 9-month clinical and MRI follow-up performed in accordance with the institutional protocol were included.

Exclusion criteria were designed to avoid confounding inflammatory or acute processes and to ensure safety and interpretability of imaging outcomes. Specifically, we excluded patients with inflammatory arthropathy or active infection, recent surgery or planned surgical procedures during follow-up, major knee instability requiring surgery, and absolute or relative contraindications to PRP injection or MRI. We also excluded patients with advanced tibiofemoral osteoarthritis on MRI, as the study focused on patellofemoral chondropathy and compositional assessment of patellofemoral cartilage.

The knee served as the unit of analysis. When both knees from a single patient met eligibility criteria, both knees were included. In the final analytic cohort, ten patients contributed bilateral knees. Due to the limited sample size and the exploratory nature of the study, no mixed-effects adjustment was applied; this limitation is explicitly acknowledged in the discussion.

### 2.3. PRP Preparation and Injection Protocol

A standardised protocol was followed in which all patients received three ultrasound-guided intra-articular platelet-rich plasma (PRP) injections at one-week intervals. PRP was prepared using a closed-kit system (Hy-Tissue PRP 20, Fidia Farmaceutici S.p.A., Abano Terme, Italy). For each session, 20 mL of peripheral venous blood was collected into a syringe containing 2 mL of 3.8% sodium citrate, then centrifuged at 1800 rpm for 8 min, yielding 4 mL of pure PRP. According to the manufacturer’s specifications, this preparation yields leukocyte-poor (pure) PRP with approximately 2.9-fold platelet enrichment and a composition of 97% platelets, 2.7% leukocytes, and 0.3% erythrocytes. PRP was activated immediately prior to injection using 10% calcium chloride. Although direct post-processing cellular counts were not available for each session, the same closed-kit system, blood volume, centrifugation parameters, and injection schedule were consistently applied to ensure procedural uniformity.

Injections were performed under sterile conditions with ultrasound guidance and delivered intra-articularly into the suprapatellar recess. Post-procedure recommendations followed standard institutional practice, including short-term relative rest and a gradual return to activity.

### 2.4. Clinical Outcomes and Definition of Response

Clinical assessment was performed at baseline and at 9 months. Pain intensity was measured using a 0–10 Visual Analogue Scale (VAS), and function was evaluated using the Kujala Anterior Knee Pain Scale (0–100). Clinical improvement was expressed as ΔVAS = VAS_baseline − VAS_9 months (positive values indicate pain improvement) and ΔKujala = Kujala_9 months − Kujala_baseline (positive values indicate functional improvement).

To complement continuous outcomes, responder status was defined a priori using minimal clinically important difference (MCID) thresholds: pain responders were those who achieved ΔVAS ≥ 1.5, and functional responders were those who achieved ΔKujala ≥ 8 [10].

### 2.5. MRI Acquisition and Image Analysis

MRI examinations were conducted at baseline and at 9 months using a 1.5 Tesla Siemens MAGNETOM SOLA system (Siemens Healthcare, Erlangen, Germany) equipped with an 18-channel knee coil. The imaging protocol comprised routine morphological sequences for structural assessment, including sagittal T1-weighted SE (TR 707 ms; TE 12 ms), sagittal proton density-weighted images with fat saturation (TR 4420 ms; TE 46 ms), T2_de3d_we_sag_iso (TR 19.88 ms; TE 7.34 ms), and T2_trufi_tra (TR 13.33 ms; TE 6 ms), as well as a quantitative cartilage mapping sequence. Quantitative T2* mapping was performed using a two-dimensional multi-echo gradient-echo sequence in the axial plane, targeting the patellofemoral joint (TR 658 ms; TE 4.35, 11.83, 19.31, 27.69, and 34.27 ms; slice thickness 3 mm; gap 0 mm; bandwidth 260 Hz/Px; field of view 160 mm; flip angle 60 degrees). Subjects were positioned supine, feet-first, with the knee in neutral rotation and approximately 15° of knee flexion. A sponge was placed to minimise motion. Quantitative maps were reconstructed using the vendor’s software environment (Syngo, Siemens Healthineers, Erlangen, Germany). Given the sensitivity of GRE-based mapping to artefacts, all examinations were visually screened, and datasets with severe motion or technical artefacts that compromised quantitative evaluation were excluded. No dedicated B0/B1 correction was applied.

**Figure 1:**
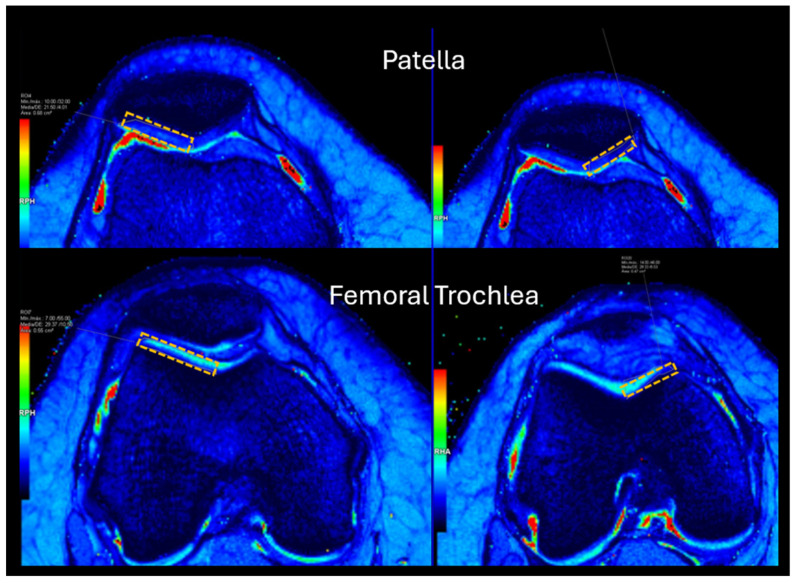
Quantitative T2* mapping requires capturing axial slices of the knee at three patellar levels and two femoral trochlear levels. Each is split into medial and lateral sections, creating six distinct regions in the patella and four in the femur. To obtain a quantitative T2* relaxation time value, a region of interest (ROI) is drawn in each area. The quantitative value of the most affected area was considered the index value (worst region).

Image analysis was conducted independently by two observers. Observer 1 provided the primary dataset for all main analyses, while measurements from Observer 2 were used to assess interobserver agreement. All image sets were anonymised and randomised to ensure blinded reading, preventing the observers from knowing patient identities or the temporal sequence (baseline or follow-up). Cartilage evaluation followed the study’s predefined regional mapping scheme, dividing the patella into six regions (P1–P6) and the femoral trochlea into four regions (F1–F4). Within each subregion, three to five standardised full-thickness regions of interest (ROIs; fixed area 3 mm^2^) were placed on magnified images (Figure 1), avoiding artefacts, synovial-fluid interfaces, and areas near subchondral bone. Region-specific T2* values were extracted at both baseline and 9 months. The main quantitative imaging variable was the baseline femoral trochlear worst-region T2*, defined as the maximum T2* value among the four femoral trochlear regions and evaluated as a candidate prognostic biomarker. Comparable worst-region values were calculated for the patella. Secondary quantitative metrics included mean compartment T2* values (averaged across regions) and longitudinal changes (ΔT2* = T2*_9 months − T2*_baseline) for both compartments. For longitudinal comparison of the most affected area, the baseline index compartment was consistently evaluated at 9 months within the same knee.

In addition to quantitative metrics, an ordinal qualitative grading of cartilage alteration was recorded on the T2* maps using a 0–4 scale (0 = normal appearance; 4 = maximal alteration). For each compartment, the worst qualitative grade (maximum across regions) was retained for analysis. Interobserver agreement for quantitative T2* metrics was assessed using ICC (2,1) absolute agreement, and agreement for ordinal qualitative grades was assessed.

### 2.6. Sample Size, Interobserver Agreement, and Statistical Analysis

An a priori sample size was estimated for the primary exploratory hypothesis of an association between baseline femoral trochlear T2* and clinical improvement. Assuming a moderate correlation (|ρ| = 0.50), a two-sided α = 0.05, and 80% power, a minimum of 30 knees was required. The final sample included 32 knees, meeting this requirement for the correlation analysis, but not for definitive multivariable prediction or reproducibility modelling.

The primary analysis evaluated continuous associations between quantitative T2* metrics and clinical changes (ΔVAS and ΔKujala) using Spearman’s rank correlation coefficient (rho). A similar continuous approach was applied to baseline mean compartment T2* values as a sensitivity analysis. Changes in VAS and Kujala scores from baseline to follow-up were assessed with the Wilcoxon signed-rank test. Responder analyses based on minimum clinically important difference (MCID) thresholds (ΔVAS ≥ 1.5; ΔKujala ≥ 8) and multivariable logistic regression models were considered secondary and exploratory, given the limited sample size. Additionally, reduced exploratory logistic sensitivity models incorporating baseline symptom severity and baseline femoral worst-region T2* were used only to evaluate the stability of the direction of association with a more parsimonious covariate structure, not to establish definitive independent predictors. Continuous variables were summarised as mean ± standard deviation (SD) or median with interquartile range (IQR), as appropriate. Due to the exploratory nature of the study and the small number of bilateral cases, mixed-effects modelling was not performed. Model diagnostics for the logistic regression analyses are provided in the Supplementary Materials. Secondary analyses were interpreted cautiously and without adjustment for multiple comparisons.

Interobserver agreement for baseline quantitative T2* metrics was quantified using ICC (2,1) (two-way random-effects model, absolute agreement). Agreement for ordinal qualitative grades was assessed using weighted κ with quadratic weights. All tests were two-sided, and *p* < 0.05 was considered statistically significant for the primary analysis. Secondary analyses were interpreted as exploratory without adjustment for multiple comparisons.

## 3. Results

### 3.1. Study Population and Clinical Outcomes

A total of 22 patients (32 knees) completed both MRI acquisitions and clinical questionnaires at baseline and at the 9-month follow-up. 10 patients contributed bilateral knees, allowing analysis of both right and left knees for each. In total, 19 right knees and 13 left knees were included. At 9 months, significant clinical improvement was observed. The median VAS decreased from 7.5 (IQR 6.5–8.5) to 5.0 (IQR 3.0–7.0), and the median Kujala score increased from 57.5 (IQR 45.0–66.0) to 68.0 (IQR 58.0–77.0) (both *p* < 0.001). Based on MCID thresholds, 22 of 32 knees (68.8%) achieved a pain response (ΔVAS ≥ 1.5), and 19 of 32 knees (59.4%) achieved a functional response (ΔKujala ≥ 8). Baseline characteristics are presented in Table 1.

### 3.2. Primary Association: Baseline Femoral T2* Values and Clinical Improvement

**Figure 2:**
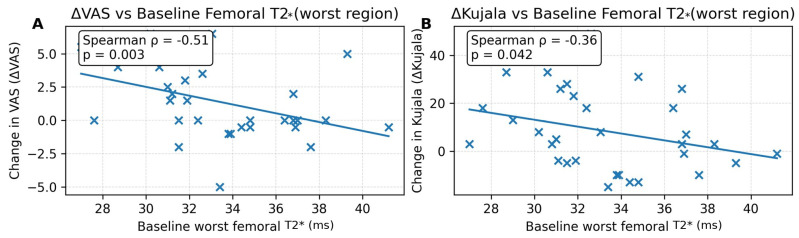
Association between baseline femoral quantitative T2* values and clinical improvement after PRP. Panel (**A**): ΔVAS (baseline VAS − 9-month VAS), Spearman’s ρ = −0.51, *p* = 0.003. Panel (**B**): ΔKujala (9-month Kujala − baseline), Spearman’s ρ = −0.36, *p* = 0.042. Dashed lines indicate MCID thresholds (ΔVAS = 1.5; ΔKujala = 8).

The primary exploratory analysis showed a significant inverse association between baseline quantitative T2* values in the femoral trochlea (worst region) and the magnitude of clinical improvement. Higher baseline femoral worst-region T2* values were associated with a lesser reduction in pain (ΔVAS: Spearman’s ρ = −0.51, *p* = 0.003) (Figure 2A). Similarly, higher baseline femoral worst-region T2* values were associated with a lesser improvement in function (ΔKujala: Spearman’s ρ = −0.36, *p* = 0.042) (Figure 2B). Given the limited interobserver reproducibility of this metric, these findings should be interpreted as an association observed in this cohort rather than evidence of a clinically validated predictive threshold.

### 3.3. Secondary Findings: Patellar T2*, Short-Term T2* Changes, and Qualitative Analysis

Unlike the femoral worst-region findings, baseline patellar T2* values in the worst region did not show a significant correlation with changes in VAS (rho = −0.18, *p* = 0.32) or Kujala scores (rho = −0.12, *p* = 0.51) (Supplementary Figure S1). Sensitivity analysis using baseline mean femoral T2* values, which showed higher interobserver agreement than worst-region values, also revealed no significant association with either ΔVAS or ΔKujala. This suggests that the observed clinical signal may be specific to the focal worst-region femoral metric and should therefore be interpreted cautiously. Detailed region-wise summaries for the patella and femoral trochlea are provided in Supplementary Tables S4 and S5.

Additionally, changes in quantitative T2* values at 9 months (ΔT2*) in both the patellar and femoral compartments were not significantly associated with clinical outcomes (all *p* > 0.1). When reported as mean regional percentage change, patellar values ranged from −3.39% to +0.73% and femoral values from −2.95% to +0.62%. None of these mean regional changes exceeded the 14% QIBA MDC95 threshold, indicating that the longitudinal T2* changes observed in this cohort are below the level generally considered necessary to distinguish true change from measurement variability with 95% confidence.

The qualitative analysis of cartilage damage using the ordinal scale (0–4) for both the patella and femur failed to discriminate between clinical responders and non-responders. For the femoral compartment, there was no significant difference in the distribution of qualitative grades between groups achieving or not achieving the MCID for Kujala (*p* = 0.581) or VAS (*p* = 0.522).

### 3.4. Exploratory Analysis of Other Potential Predictors

Exploratory bivariate and multivariable analyses were conducted to identify additional factors associated with achieving a clinically meaningful response (MCID). Given the limited sample size, the event-per-variable ratio, and the wide confidence intervals, these models functioned exclusively as hypothesis-generating analyses rather than providing definitive causal or predictive inference. In reduced exploratory sensitivity models that included only baseline symptom severity and baseline femoral worst-region T2*, the inverse direction of association for femoral T2* persisted. However, these parsimonious models also lacked sufficient power for definitive multivariable inference.

Regarding pain response (MCID on VAS), bivariate analysis indicated that a higher baseline VAS score was associated with an increased likelihood of response (*p* = 0.00015). Conversely, a history of previous intra-articular injections (hyaluronic acid or glucocorticoid) was inversely associated with response (OR = 0.17, *p* = 0.027). In the multivariable logistic regression model, only baseline VAS pain remained statistically significant (adjusted OR = 14.54, 95% CI: 1.26–167.3, *p* = 0.032) (Supplementary Table S1). This estimate should not be overinterpreted given the small cohort size and wide confidence interval.

For functional response (MCID on Kujala), no variables reached statistical significance in bivariate analysis. In the multivariable model, male sex was associated with a higher probability of response (adjusted OR = 33.8, 95% CI: 1.63–701.1, *p* = 0.023). However, the extremely wide confidence interval indicates substantial model instability; therefore, this finding should be regarded only as exploratory and should not be considered a reliable independent predictor (Supplementary Table S2).

Other variables, including age, BMI, platelet count, platelet concentration, and morphological parameters of the patellofemoral joint (e.g., patellar tilt, trochlear angle), showed no consistent or significant associations with clinical response in these exploratory models.

Interobserver agreement for worst-region T2* was limited, particularly for the femoral compartment (femur ICC (2,1) 0.37; patella ICC (2,1) 0.47), and improved for mean T2* values (femur ICC (2,1) 0.54; patella ICC (2,1) 0.65). Weighted κ for worst-grade qualitative scoring was 0.62 for patella and 0.04 for femur (Supplementary Table S3). These results indicate that the focal worst-region metric is more vulnerable to reader-dependent ROI placement and local cartilage heterogeneity than mean compartmental T2* values.

## 4. Discussion

To our knowledge, this retrospective study based on routine clinical practice is among the first to explore baseline quantitative T2* mapping of the femoral trochlea as a candidate prognostic biomarker associated with clinical response to intra-articular PRP in patellofemoral chondropathy. The principal finding was a significant inverse association between baseline femoral worst-region T2* values and the magnitude of improvement in both pain and function. This suggests that the pre-treatment compositional status of the femoral cartilage may be related to subsequent clinical response. However, because the worst-region metric showed limited interobserver reproducibility, this association should be considered preliminary and hypothesis-generating rather than evidence of a clinically validated predictor.

PRP is a biologically active autologous product containing a complex mixture of growth factors, cytokines, and bioactive mediators that can modulate the joint microenvironment, including synovial inflammation, nociceptive signalling, and chondrocyte metabolism [11]. From a cartilage “molecular imaging” perspective, quantitative T2* mapping captures matrix ultrastructure—primarily collagen organization and hydration—thereby reflecting biochemical/cartilage-compositional deterioration that may precede overt morphological breakdown. The observed association between higher baseline femoral trochlear worst-region T2* values and poorer clinical response is compatible with the concept of a possible “window of opportunity”, in which relatively preserved cartilage matrix may be more likely to accompany symptomatic and functional improvement after biologic treatment. This interpretation remains speculative and requires external validation.

The compartment-specific nature of this association is noteworthy but should also be interpreted cautiously. While the femoral trochlea showed an association with outcomes in the worst-region analysis, baseline patellar T2* values did not correlate significantly with clinical improvement. This divergence may reflect differences in biomechanical roles within the patellofemoral joint, differences in regional cartilage loading, or greater measurement variability in focal ROI-based assessments. The finding may refine the interpretation of prior work, such as the study by Cobianchi Bellisari et al. [7], which reported patellofemoral T2* changes after PRP, by suggesting that the femoral compartment merits specific evaluation in future studies.

Conversely, we did not observe a meaningful association between short-term changes in T2* mapping (ΔT2* at 9 months) and clinical improvement. This dissociation between imaging and patient-reported outcomes is not unexpected. The mean regional percentage changes observed in this cohort were small (patella: −3.39% to +0.73%; femur: −2.95% to +0.62%) and did not exceed the 14% QIBA MDC95 threshold [4]. Therefore, these longitudinal T2* variations should not be interpreted as definitive evidence of short-term structural cartilage change and may primarily reflect measurement variability. The symptomatic benefits of PRP, when present, may be mediated more by modulation of synovial inflammation, nociceptive signalling, or joint homeostasis than by rapid quantifiable cartilage matrix remodelling within this timeframe. This concept is supported by trials such as RESTORE, in which PRP provided pain relief without slowing structural cartilage loss [12]. The lack of predictive value of qualitative ordinal grading further underscores the need for reproducible quantitative workflows before compositional MRI metrics can be translated into clinical decision-making.

Our exploratory analyses, while constrained by sample size, point to the multifactorial nature of treatment response. The association between higher baseline pain intensity and achieving a pain MCID is clinically plausible because patients with worse baseline symptoms have a larger potential margin for improvement. The inverse association between prior intra-articular injections and pain response may identify a subgroup with more refractory or chronic pathology, although this interpretation remains exploratory. Baseline femoral T2* did not remain a definitive independent predictor in multivariable MCID models, which is plausibly explained by limited power after dichotomising outcomes, covariance with baseline symptom severity, and model instability in a small cohort. These clinical factors, together with the imaging association observed in the primary analysis, support the need for future integrated prediction models combining clinical, morphological, and compositional data [13].

Several limitations of this study should be acknowledged. First, the single-centre retrospective observational design without a control group prevents causal inference and does not permit attribution of symptomatic improvement specifically to PRP. Second, because the knee was the unit of analysis, inclusion of 10 bilateral patients introduces potential within-patient correlation. Mixed-effects modelling was not performed due to the small exploratory cohort, which may have influenced variance estimates. Third, interobserver reproducibility was limited for worst-region quantitative metrics, especially in the femoral compartment, and higher for mean T2* values. This indicates that extreme-value summaries are sensitive to reader-dependent ROI placement and local cartilage heterogeneity. Intraobserver reproducibility was not assessed in this retrospective routine-practice dataset, further limiting precision assessment. Consequently, the association observed for baseline femoral worst-region T2* should be considered hypothesis-generating and requires validation in larger cohorts, with repeated reads and more standardised or semiautomated segmentation approaches. Fourth, direct post-processing cellular quantification of each PRP injectate was not available, so biological characterisation relied on a standardised closed preparation system and manufacturer data rather than laboratory verification of each session. Fifth, the 9-month follow-up period may be insufficient to capture slow compositional remodelling, and the modest longitudinal T2* changes observed were below QIBA-related minimum detectable thresholds; specifically, the mean regional percentage changes remained below the 14% QIBA MDC95 threshold. Finally, absolute T2* values are dependent on MRI hardware and sequence parameters; therefore, the specific numeric thresholds proposed require external validation and protocol harmonisation prior to clinical implementation.

Despite these limitations, the findings may have potential clinical relevance if externally validated. Baseline femoral T2* mapping could contribute to future patient stratification and shared decision-making by identifying cartilage compositional profiles associated with greater or lesser symptomatic improvement after PRP. However, the present data do not support immediate clinical implementation or a validated treatment-selection threshold. Future studies should prioritise larger multicentre cohorts, control groups, adjustment for bilateral knees, repeated intra- and interobserver readings, and standardised or semiautomated segmentation workflows before this candidate biomarker can be translated into routine practice.

## 5. Conclusions

In this small retrospective exploratory cohort, baseline quantitative T2* values in the most affected region of the femoral trochlea were significantly associated with the degree of clinical improvement following platelet-rich plasma (PRP) treatment in patients with patellofemoral chondropathy. In contrast, baseline patellar T2* measurements, baseline mean femoral T2* sensitivity analyses, and short-term changes in T2* did not demonstrate similar associations. The worst-region femoral T2* metric should therefore be regarded as a candidate prognostic biomarker rather than a clinically validated predictor. Because interobserver reproducibility of worst-region measurements was limited and longitudinal ΔT2* values remained below QIBA minimum detectable change thresholds, these preliminary hypothesis-generating findings require validation in larger, controlled, and methodologically standardised studies before routine clinical implementation.

## Figures and Tables

**Table 1:** Baseline characteristics of the study population (*n* = 32 knees).

Characteristic	Overall
Number of knees	32
Number of patients	22
Male sex	13 (40.6%)
Age (years)	43.1 ± 11.0
BMI (kg/m^2^)	26.1 ± 4.5
Right knee	19 (59.4%)
Left knee	13 (40.6%)
Patients with bilateral involvement	10
Prior intra-articular injection (any, at least 6 months ago)	15 (46.9%)
Prior hyaluronic acid injection (at least 6 months ago)	12 (37.5%)
Prior glucocorticoid injection (at least 6 months ago)	5 (15.6%)
Baseline VAS (0–10)	7.5 (7.0–8.0)
Baseline Kujala (0–100)	51.0 (44.8–65.2)
Baseline femoral T2* (ms), worst region	31.9 ± 5.0
Baseline patellar T2* (ms), worst region	30.8 ± 6.0

Continuous variables are reported as mean ± SD unless otherwise indicated. VAS and Kujala are reported as median (IQR).

## Data Availability

Data are available from the corresponding author upon reasonable request.

## Supplementary Materials

The following supporting information can be downloaded at: https://www.mdpi.com/article/10.3390/jcm15114324/s1, Figure S1: Baseline Patellar T2* and clinical change after PRP; Figure S2: Bland-Altman analysis of baseline T2* measurements; Table S1: Multivariable logistic regression model for pain response (MCID on VAS, ΔVAS ≥ 1.5); Table S2: Multivariable logistic regression model for functional response (MCID on Kujala, ΔKujala ≥ 8); Table S3: Interobserver agreement for baseline quantitative and qualitative T2 mapping metrics; Table S4: Observer 1 patellar regional quantitative T2* values at baseline and 9 months (P1–P6); Table S5: Observer 1 femoral trochlear regional quantitative T2* values at baseline and 9 months (F1–F4).
