# Supplementary material for: Association of Baseline Femoral Trochlear T2* Mapping with Clinical Response to Platelet-Rich Plasma in Patellofemoral Chondropathy: A Retrospective Exploratory Study
Source: J Clin Med. 2026 Jun 3;15(11):4324. doi: 10.3390/jcm15114324 (PMC13257490; doi:10.3390/jcm15114324)
Supplement: Supplementary file 1 [file jcm-15-04324-s001.zip › jcm-4222480-supplementary.pdf]

Journal of
Clinical Medicine

MDPI

Supplementary Materials

Supplementary Figure S1. Baseline patellar T2 and clinical change after PRP

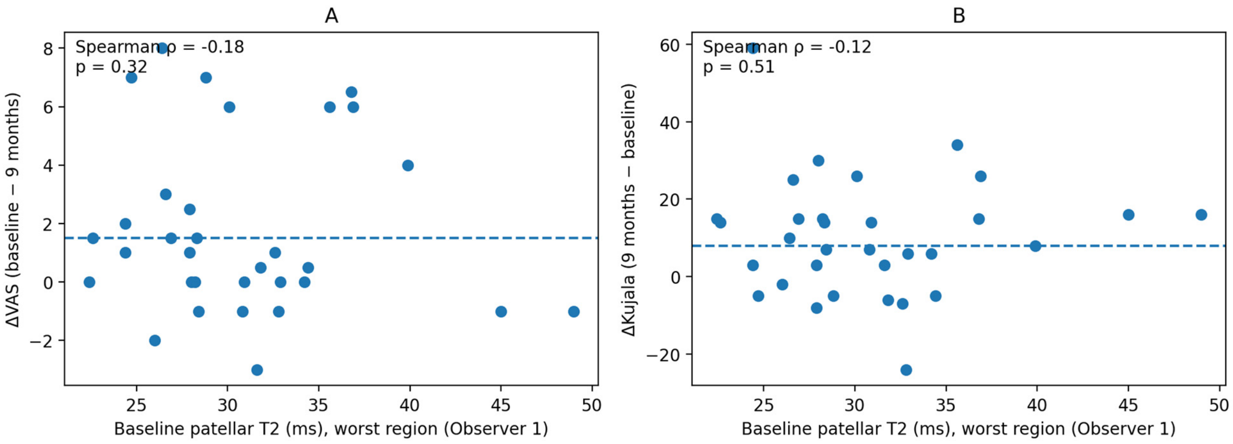

Figure S1. Lack of association between baseline patellar T2* values (worst region; Observer 1) and clinical improvement after PRP. Panel A shows baseline patellar T2* vs ΔVAS with the MCID threshold (ΔVAS ≥ 1.5) indicated by a dashed line. Panel B shows baseline patellar T2* vs ΔKujala with the MCID threshold (ΔKujala ≥ 8) indicated by a dashed line.

Table S1. Multivariable logistic regression model for pain response (MCID on VAS, ΔVAS ≥ 1.5).

Predictor Variable	B Coefficient (SE)	Adjusted OR (95% CI)	p-Value
Baseline VAS score (per point)	2.721 (1.245)	15.2 (1.31–176.8)	0.029
Previous intra-articular injections (Yes vs No)	−1.833 (0.838)	0.16 (0.03–0.82)	0.028
Age (per year)	−0.039 (0.041)	0.96 (0.89–1.04)	0.340
Sex (Male vs Female)	0.854 (0.867)	2.35 (0.43–12.86)	0.325
BMI (per kg/m²)	−0.101 (0.095)	0.90 (0.75–1.09)	0.287
Baseline femoral T2 (per ms)	−0.059 (0.060)	0.94 (0.84–1.06)	0.326
Constant	−3.024 (3.845)	0.05	0.431

Model statistics: Nagelkerke $R^2 = 0.47$; Hosmer–Lemeshow test $p = 0.65$; overall classification accuracy = 78.1%.

Table S2. Multivariable logistic regression model for functional response (MCID on Kujala, ΔKujala ≥ 8).

Predictor Variable	B Coefficient (SE)	Adjusted OR (95% CI)	p-Value
Sex (Male vs Female)	3.450 (1.527)	31.5 (1.58–628.4)	0.024
Baseline Kujala score (per point)	−0.043 (0.046)	0.96 (0.88–1.05)	0.351
Age (per year)	0.025 (0.047)	1.03 (0.94–1.12)	0.598
BMI (per kg/m²)	0.118 (0.106)	1.13 (0.91–1.39)	0.266
Previous intra-articular injections (Yes vs No)	−0.921 (0.974)	0.40 (0.06–2.68)	0.345
Baseline femoral T2 (per ms)	−0.078 (0.064)	0.93 (0.82–1.05)	0.223
Constant	−5.641 (5.102)	0.004	0.269

Model statistics: Nagelkerke $R^2 = 0.39$; Hosmer–Lemeshow test $p = 0.82$; overall classification accuracy = 71.9%.

Table S3. Interobserver agreement for baseline quantitative and qualitative T2 mapping metrics.

**Metric (Baseline)**	**Statistic**	**Estimate (95% CI)**
Femoral trochlear T2 (worst region)	ICC(2,1)	0.37 (−0.08 to 0.76)
Patellar T2 (worst region)	ICC(2,1)	0.47 (0.09 to 0.72)
Femoral trochlear T2 (mean)	ICC(2,1)	0.54 (0.27 to 0.76)
Patellar T2 (mean)	ICC(2,1)	0.65 (0.37 to 0.83)
Patellar qualitative grade (worst)	Weighted κ	0.62 (0.27 to 0.86)
Femoral qualitative grade (worst)	Weighted κ	0.04 (0.00 to 0.08)

Note: ICC(2,1) = intraclass correlation coefficient (absolute agreement, two-way random effects); κ = kappa coefficient with quadratic weighting.

Supplementary Figure S2. Bland-Altman analysis of baseline T2* measurements

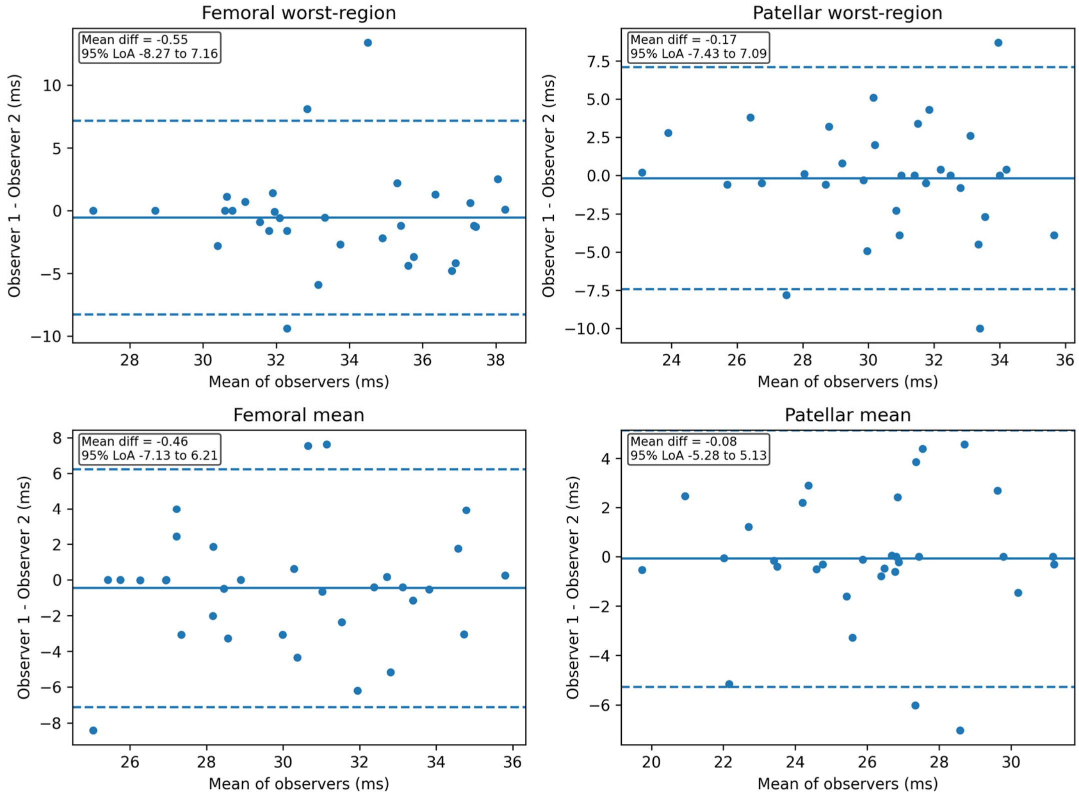

Figure S2. Bland-Altman plots for baseline interobserver agreement in worst-region and mean T2* measurements. Panels show femoral worst-region, patellar worst-region, femoral mean, and patellar mean values. Solid lines indicate the mean interobserver difference and dashed lines the 95% limits of agreement.

Table S4. Observer 1 patellar regional quantitative T2* values at baseline and 9 months (P1-P6).

Region	Baseline (ms)	9 months (ms)	ΔT2* (ms)
P1	27.73 ± 4.40	27.83 ± 4.94	0.09 ± 3.47
P2	25.82 ± 3.57	25.60 ± 3.66	-0.22 ± 3.43
P3	26.23 ± 4.23	25.34 ± 4.09	-0.89 ± 3.13
P4	25.30 ± 5.20	25.03 ± 4.18	-0.27 ± 3.76
P5	27.23 ± 4.37	27.43 ± 4.87	0.20 ± 4.48
P6	24.02 ± 4.73	23.51 ± 4.12	-0.51 ± 3.56

Values are presented as mean ± SD. Regional summaries correspond to Observer 1 measurements.

Table S5. Observer 1 femoral trochlear regional quantitative T2* values at baseline and 9 months (F1-F4).

Region	Baseline (ms)	9 months (ms)	ΔT2* (ms)
F1	32.40 ± 3.40	32.10 ± 3.88	-0.29 ± 4.00
F2	29.87 ± 3.64	29.59 ± 4.26	-0.27 ± 4.74
F3	29.96 ± 4.67	29.07 ± 4.27	-0.88 ± 4.53
F4	27.55 ± 5.47	27.72 ± 4.80	0.17 ± 3.44

Values are presented as mean ± SD. Regional summaries correspond to Observer 1 measurements.

Disclaimer/Publisher's Note: The statements, opinions and data contained in all publications are solely those of the individual author(s) and contributor(s) and not of MDPI and/or the editor(s). MDPI and/or the editor(s) disclaim responsibility for any injury to people or property resulting from any ideas, methods, instructions or products referred to in the content.

https://doi.org/10.3390/jcm15114324
